# Balancing the risks of rheumatic heart disease and antimicrobial resistance in South Africa: Insights from a local workshop

**DOI:** 10.4102/safp.v68i1.6331

**Published:** 2026-09-17

**Authors:** Oliver van Hecke, Renier Coetzee

**Affiliations:** 1 Department of Public Health and Primary Care, Faculty of Medicine, Ghent University, Ghent, Belgium; 2 School of Public Health, Faculty of Community and Health Sciences, University of the Western Cape, Cape Town, South Africa

**Keywords:** antibiotic stewardship, rheumatic heart disease, sore throat, antibiotic prescribing, tonsilitis

## Abstract

There is a significant burden of rheumatic heart disease (RHD) in South Africa, where timely diagnosis and treatment of Group A *Streptococcus* with antibiotics have made significant inroads over the last decade. However, despite the well-established benefits of penicillin for RHD, concerns about antimicrobial resistance have intensified debates around antibiotic stewardship, access and long-term strategies.

**Contribution:**

We outline the findings of a transdisciplinary workshop with key stakeholders in South Africa. A careful balance is needed to achieve the dual goals of protecting vulnerable populations from RHD while upholding responsible and sustainable antibiotic use. Key areas that need to be addressed include the clinical assessment of sore throat, harmonisation of current treatment guidelines in South Africa and policy adaptations.

## Introduction

### The challenge

South Africa has a particularly high burden of infectious diseases. Group A *Streptococcus* (GAS, *Streptococcus pyogenes*) causes a wide spectrum of clinical illnesses, including sore throat (pharyngitis), scarlet fever and skin infections (e.g. impetigo). In a small proportion of cases, these infections can trigger the onset of acute rheumatic fever (ARF) and its major sequela, rheumatic heart disease (RHD).^1^ Both ARF and RHD relate to poverty and are regarded as classic diseases of social injustice.^2^ These conditions are also intricately entwined with at least three Sustainable Development Goals (SDGs) (SDG 1 No Poverty; SDG 3 Good Health and Well-being; SDG 6 Clean Water and Sanitation).^3^ Ninety-five per cent of the disease burden from RHD is in low- and middle-income countries and in disadvantaged populations in high-income countries where it continues to have a significant impact on the health of children and young adults.^4,5^

The primary prevention of ARF encompasses the timely diagnosis and adequate treatment of superficial GAS infections.^6^ In the South African public healthcare sector, which treats more than 80% of the population,^7^ these infections present first in primary care and are largely managed entirely by nurses in the community. Based on published data in South Africa until 2014, the overall crude incidence of symptomatic RHD in adults is estimated at 24.7 per 100 000 (95% confidence interval [CI] 22.1–27.4) population per annum among adults (≥ 13 years) in Soweto.^8^ The prevalence of asymptomatic echocardiographic RHD in schoolchildren was 20.2 cases per 1000 children (95% CI 15.3–26.2) in Cape Town.^9^

### The dilemma

Despite significant shifts in our understanding of the disease pathogenesis of ARF, and access to high-quality penicillin antibiotics in South Africa, many unanswered questions remain. For example, are the right children (at risk for complications of GAS) with acute (bacterial) sore throat being identified and treated with penicillin? In a recent prospective observational study (PRINS study) in the Cape Town Metropole, nine out of every 10 patients (adults and children) were prescribed a penicillin antibiotic for acute sore throat or pharyngitis,^10^ compared to local prospective data from the same region, suggesting that one in five children with symptomatic sore throat had a positive GAS culture.^11^ Identifying ‘at risk’ children is clinically challenging as children can be either asymptomatic or have so mild symptoms that medical attention is never sought.

Likewise, most children with symptomatic sore throat will not have GAS and prescribing antibiotics to these children is problematic for many reasons, including microbiome dysbiosis and driving the development of resistant infections.

Accurate primary care data are needed on the disease epidemiology, diagnostic and treatment practices of acute sore throat infections in publicly funded Primary Health Care facilities. For example, although several clinical decision tools exist to help predict the likelihood of a streptococcal bacterial infection,^11,12,13,14^ we do not know whether primary care clinicians routinely use them in South Africa. We therefore set out to convene a transdisciplinary stakeholder group and explore the primary care management of uncomplicated sore throat, including GAS sore throat, in the context of RHD in South Africa.

## Methods

The authors organised a local workshop to run in October 2025 at Groote Schuur Hospital, Cape Town. Key stakeholders were invited: members of the primary care clinical team (doctors, nurse prescribers pharmacists) working in Community Health Centres (CHCs) or Community Day Centres (CDCs), microbiologists, infectious diseases specialists, researchers, public health doctors and policymakers. The overall aim of the workshop was to identify areas where and how we could improve the management of sore throat in children in publicly funded Primary Health Care Facilities in the Western Cape.

Participants were briefed about the scope of the workshop. They initially listened to individual presentations (including Questions and Answers) given by representatives of each topic domain, on:

The burden of RHD in South Africa, the current approach to sore throat in children in the context of moderate or high risk for rheumatic fever, reflections on observational data on sore throat management in children in the Western Cape Metro (PRINS Study), and comparison with the management of sore throat in children in a setting with a low risk for rheumatic fever.

Participants were then divided into smaller groups to reflect on the presentations and asked to consider: (1) whether the correct children (at risk) are being treated with antibiotics; and (2) the potential stewardship strategies that could be implemented to optimise the treatment of acute sore throat infections in children. Participants then fed back to the wider group for further discussion. Key suggestions for interventions and opportunities were debated.

### Ethical consideration

We confirm that ethical approval was not needed or required for this study.

## Results

Twenty-one participants contributed to the workshop (four family physicians, one medical officer, three researchers from Children’s Heart Disease Research Unit, two Knowledge Translation scientists, five public health and/or policymakers within the Western Cape Department of Health, one infectious diseases physician, four antimicrobial stewardship pharmacists, one PhD student).

There were two main areas where participants thought that improvements could be made, namely (1) in the clinical assessment and diagnosis of acute sore throat and (2) in the treatment thereof.

Although there have been significant inroads into tackling RHD in South Africa, there currently is no national patient registry. Participants voiced that there is a need to better identify children with sore throat that would benefit from antibiotic treatment in South Africa. Although clinician participants acknowledged that most sore throat infections are viral in this age group, they were mindful that some frontline clinicians would be wary of missing a diagnosis of GAS. Likewise, there was limited oversight of antibiotic prescribing practices, especially where antibiotics were dispensed within the consultation room and no routine antibiotic audit feedback loop within facilities.

They wanted to see refresher courses on the management of sore throat (including GAS sore throat) offered to frontline clinicians. Currently there is no system in place to flag and follow up patients with potential GAS sore throat. Although participants saw the value of GAS point-of-care test (POCT) in ruling out the presence of GAS, testing at this scale would be prohibitive in the public sector without more evidence. Most participants were unaware of available clinical decision support tools (e.g. FeverPAIN, Centor) and the specific decision support tool developed in Cape Town (The Cape Town Clinical Decision Rule) and did not incorporate these in their clinical decision-making.

Participants remarked that the various South African treatment guidelines^15,16,17,18^ aimed at primary care clinicians were not aligned (see Table 1). Guidelines had different interpretations of the symptom criteria for GAS sore throat. Specifically, there was no weighting of GAS sore throat criteria to indicate the probability of GAS sore throat in any of the guidelines. Participants perceived that without putting any weight on clinical symptoms, many clinicians would risk misinterpreting the guideline and prescribe an antibiotic.

**TABLE 1:** Summary of South African treatment guidelines for acute sore throat in children (differences are in italics).

Guideline	Excerpt from guidance related to acute sore throat in children
The Standard Treatment Guidelines and the Essential Medicines List (Primary Health Care Level) 2024 edition^15^	Antibiotics are not required for all patients with a sore throat. Antibiotics to eradicate streptococci must be given to patients presenting with a sore throat who are at risk for rheumatic fever (3–21 years of age) if they have: » Enlarged tonsils PLUS at least one of the following criteria: - Exudates on their tonsils - No cough - No runny nose
PACK Primary Care Guide for the Child 2026, Western Cape Edition^16^	Are there swollen tonsils and any of: pus/white patches on tonsils or no cough or no runny nose? *Child may also have a generalised red rash, red spots in back of throat or a* red tongue with bumps (*strawberry tongue*).
Integrated Management of Childhood Illness 2022^17^	If the child is 3 years old or older, ask: Does the child have a sore throat? Enlarged tonsils with white or yellow exudate AND One or more of the following: - No runny nose - No cough - *Scarlatiniform rash*
Primary Care Nurse Specialist Guide^18^	Streptococcal pharyngitis: Tonsil enlargement, tonsillar exudate, *tender and enlarged anterior cervical nodes*, absence of cough. High temperature (more than 38°C) is also an important finding.

Note: Please see the full reference list of Van Hecke O, Coetzee R, Balancing the risks of rheumatic heart disease and antimicrobial resistance in South Africa: Insights from a local workshop. S Afr Fam Pract. 2026;68(1), a6331. https://doi.org/10.4102/safp.v68i1.6331.

In terms of treatment, all guidelines mentioned the single-dose benzathine benzylpenicillin IM because of its greater efficacy compared to oral antibiotics.^19^ However, participants voiced that intramuscular penicillin was rarely given in clinical practice because of out-of-stock issues and difficulties procuring medicines. Instead, oral amoxicillin or phenoxymethylpenicillin was given for GAS sore throat for 10 days. However, this extended oral antibiotic regimen then risks patients not completing the treatment course.

## Discussion

This workshop provided critical perspectives from transdisciplinary stakeholders to identify areas where and how the management of sore throat in children could be optimised in the unique context of RHD in South Africa.

Clinically, identifying children (at risk for complications of GAS) with apparently uncomplicated sore throat remains challenging. The use of a Strep A POCT to rule in symptomatic GAS sore throat has its merits in regions with a low risk for rheumatic fever. In such regions, the use of POCT to detect GAS could reduce overall antibiotic prescribing by approximately 25% in patients with acute sore throat and, perhaps more importantly, target antibiotic prescribing to patients harbouring GAS.^20^

In a setting like South Africa with a relatively higher risk for rheumatic fever, the use of POCT would seem obvious to rule out GAS, except for the prohibitive cost in the public sector. Hence, including locally validated clinical scoring tools such as the Cape Town Clinical Decision Rule in guidelines would offer a potential solution (see Table 2).

**TABLE 2:** Scoring system of the Cape Town clinical decision rule developed to calculate the risk of streptococcal pharyngitis positivity in an individual participant.

Clinical variable	Allocated point score	Estimated probability of GAS (%) based on PPV*
Absence of cough	+1	Low risk score 0 to 1 → ~20% – 25% probability of isolating *Streptococcus*
Absence of rhinorrhoea	+1	
Presence of tonsillar swelling	+2	Moderate risk score 2 to 3 → ~23% – 25% probability of isolating *Streptococcus*
Presence of tonsillar exudate	+1	Highest risk score 4 to 5 → ~30% – 36% probability of isolating *Streptococcus*

*Source:* Engel ME, Cohen K, Gounden R, et al. The Cape Town clinical decision rule for Streptococcal Pharyngitis in children. Pediatr Infect Dis J. 2017;36:250–255. https://doi.org/10.1097/INF.0000000000001413

PPV, positive predictive value.

* , The baseline (pre-test probability) GAS prevalence in the Engel et al. 2017 study was 21%.^11^

For policymakers and guideline developers, our findings support the need for respective guidelines used in primary care to be harmonised. The Standard Treatment Guidelines and the Essential Medicines List were seen as the main guideline which other guidelines tended to follow. However, the clinical symptom criteria for GAS sore throat (mentioned in all guidelines) need to reflect the scoring or likelihood (probability) of a sore throat being a result of a GAS bacterial infection. Validated local studies like the Cape Town Clinical Decision Rule study have this information. There is good evidence that applying specific decision support tools can optimise the treatment for uncomplicated sore throat.^14^

In terms of further research, our findings suggest the need to test the implementation of a locally validated clinical decision support tool and/or Strep A POCT to improve symptoms and reduce antibiotic use in uncomplicated sore throat without any negative consequences. Evidence from Europe suggests that many children with RHD never sought medical care as their symptoms were non-severe. Hence, longitudinal case-control studies are needed to explore which children with a GAS or bacterial throat infection go on to develop RHD in South Africa.

## Conclusion

Our transdisciplinary workshop raised important stakeholder perspectives on the clinical assessment, diagnosis and treatment of acute sore throat, which have implications for guideline developers, primary care clinicians and researchers in the Southern African region.
